# Stress, Sleep and Psychological Impact in Healthcare Workers During the Early Phase of COVID-19 in India: A Factor Analysis

**DOI:** 10.3389/fpsyg.2021.611314

**Published:** 2021-02-25

**Authors:** Seshadri Sekhar Chatterjee, Madhushree Chakrabarty, Debanjan Banerjee, Sandeep Grover, Shiv Sekhar Chatterjee, Utpal Dan

**Affiliations:** ^1^Department of Psychiatry, Diamond Harbour Government Medical College and Hospital (DHGMC), Diamond Harbour, India; ^2^Department of Psychiatry, National Institute of Mental Health and Neurosciences (NIMHANS), Bengaluru, India; ^3^Department of Psychiatry, Post Graduate Institute of Medical Education and Research (PGIMER), Chandigarh, India; ^4^Department of Microbiology and In-charge, COVID Testing Unit, Diamond Harbour Government Medical College and Hospital (DHGMC), Diamond Harbour, India; ^5^Department of Anatomy and Principal, Diamond Harbour Government Medical College and Hospital (DHGMC), Diamond Harbour, India

**Keywords:** COVID-19, healthcare workers, physicians, perceived stress, sleep, psychological wellbeing

## Abstract

**Background:** Risks to healthcare workers have escalated during the pandemic and they are likely to experience a greater level of stress. This cross-sectional study investigated mental distress among healthcare workers during the early phase of Coronavirus disease-2019 (COVID-19) outbreak in India.

**Method:** 140 healthcare workers of a tertiary care hospital in India were assessed for perceived stress and insomnia. A factor analysis with principal component method reduced these questions to four components which were categorized as insomnia, stress-related anxiety, stress-related irritability, and stress-related hopelessness. Further statistical analyses were done on these factor scores to identify the predictors and investigate the differences between the different categories of healthcare workers.

**Result:** Doctors had the highest level of anxiety among the healthcare workers. Both doctors and nurses perceived a greater level of irritability than the other HCWs. Compared to doctors and nurses, other HCWs were more likely to experience insomnia. Lower age, higher education, female gender, and urban habitat were associated with greater perception of anxiety. Older age, being quarantined, and single marital status were the significant predictors of irritability. Female gender, single marital-status, and higher number of medical ailments contributed to perceived hopelessness. Quarantine significantly predicted insomnia.

**Conclusion:** Different categories of healthcare workers are experiencing varied mental health problems owing to their heterogeneous socio-demographic backgrounds. Tailored and personalized care, as well as policies, might help in alleviating their problems. Further research is warranted to explore the psychological distress and remedies among these frontline workers during and after the ongoing pandemic crisis.

## Introduction

Coronavirus disease-2019 (COVID-19) has created an unprecedented situation worldwide and has set forth an array of challenges before us—medical, ethical, social, and organizational (Mukherjee et al., 2020). Health care workers (HCWs) are bound by ethics to provide support to patients (Neto et al., 2020). Adhering to medical ethics, HCWs across the world are putting their fullest effort to cope with the pandemic and save lives. However, they are not immune to infection risk. Consequently, HCWs are equally vulnerable to infection as the rest of the population. In fact, the frontline workers are at a greater risk than the general population. Previous statistics clearly indicate that HCWs make a significant portion of the infected cases (Simonds and Sokol, 2008).

Owing to increased risk of infection, duty toward patients might tussle with self-preservation and protection of loved ones thereby increasing stress and anxiety of HCWs (Tam et al., 2004; Ehrlich et al., 2020). Increased duty hours and disrupted biological rhythm during the quarantine might lead to insomnia (Liu et al., 2020). Inadequate supply of personal protective equipment, problematic media coverage and stigma might exacerbate stress (Lai et al., 2020; Malathesh et al., 2020; Menon et al., 2020). In a recent review of six studies, Spoorthy et al. (2020) reported that “HCW are encountering a considerable degree of stress, anxiety, depression, insomnia due to the COVID-19 pandemic.” Apart from doctors, people working in healthcare facilities such as nurses, ward staff, cleaning staff, porters, and administrative staff are also variably vulnerable (Que et al., 2020) and might face mental health problems. People working in certain specialties such as a respiratory ward, infectious diseases ward or critical care ward are subject to greater risk and might be under greater stress.

In a recent review of 43 studies on the psychological impact of COVID-19, Vindegaard and Benros (2020) stated that several factors might be associated with a higher risk of psychological distress among healthcare workers as well as the general public. In fact, the female gender (Mazza et al., 2020; Zhang et al., 2020b), lower educational level (Gao et al., 2020; Mazza et al., 2020), lack of family/social support (Cao et al., 2020; Du et al., 2020), living in urban areas (Gao et al., 2020; Özdin and Bayrak Özdin, 2020), poor social capital and/or unstable income (Cao et al., 2020; Xiao et al., 2020), higher social media exposure (Gao et al., 2020), previous experience of distressful life events (Mazza et al., 2020), lack of preparedness (Du et al., 2020), not adhering to safety or precautionary measures (Wang et al., 2020a), poor self-rated health (Gao et al., 2020; Wang et al., 2020a,b), having a history of chronic illness including psychiatric disorder and substance abuse (Mazza et al., 2020; Özdin and Bayrak Özdin, 2020; Wang et al., 2020b), having a COVID-19 infected friend or relative (Cao et al., 2020; Du et al., 2020; Mazza et al., 2020; Özdin and Bayrak Özdin, 2020), poor sleep quality (Du et al., 2020), higher perceived stress (Du et al., 2020), working in frontline (Giorgi et al., 2020; Lai et al., 2020; Lu et al., 2020), working in a secondary hospital (Lai et al., 2020), intermediate position in job (Lai et al., 2020), seniority in the workplace (>10 years) (Lai et al., 2020) etc. were frequently associated with increased risk of psychological distress. However, there are several inconsistencies and researchers are still not unequivocal regarding these associations. For, example, while several studies identified living in urban areas as a potent risk factor for psychological distress (Gao et al., 2020; Özdin and Bayrak Özdin, 2020), few others reported that living in rural areas could increase the risk (Cao et al., 2020; Zhang et al., 2020b). It may be noted here that Gao et al. (2020) studied the general Chinese population and Zhang et al. (2020b) studied the health care workers of China. Thus, risk factors may vary in different populations and studies focused on different target populations are needed for proper identification of the risk factors and subsequent redemption.

India with its several densely populated states, shortage of medical professionals, inadequate equipment, scarcity of health centers, the paucity of testing facilities, sparse surveillance, and poor awareness among masses, failed to contain the disease (Kumar et al., 2020). Consequently, the pressure on the health system mounted. The Government of India ordered a nationwide lockdown for 21 days On March 24, 2020. The lockdown was further extended with conditional relaxations. The pandemic coupled with lockdown made a deep impact on the socio-economic fabric as well as the mental health conditions of the people. Apprehensions and anguish transformed into fear and stigma toward COVID-19 patients as well as fighters (Bagcchi, 2020). In India, HCW dealing with COVID-19 patients faced considerable social rejection and ostracism. Forceful eviction from temporary residence by house owners, discrimination, violent attacks in public places, and public transports posed threat to their lives. Social stigma against COVID-19 made the difficult situation worse for HCWs. Inadequate numbers of public health care centers along with the escalating COVID-19 treatment expenses in the private health care centers worsened the situation (Mitra, 2020). The already dwindling patient-doctor relationship (Tripathi et al., 2019) reached a worrying level of distrust. Health care workers in general and public health care workers, in particular, suffered acute helplessness. Stigma, work overload, shortage of equipment, dying patients, distrust, concern for personal safety, and safety of the family members pushed them into mental turmoil.

Recent studies on Indian doctors reported significant mental health problems due to COVID-19 (Chatterjee et al., 2020; Khanam et al., 2020; Podder et al., 2020). 52.8% of the health care workers in India were reported to have COVID-19 pandemic-related burnout (Kulkarni et al., 2020). In another study, 73.9 and 30% of the dermatologists in India were found to experience stress and insomnia, respectively due to the pandemic (Bhargava et al., 2020). This is quite in line with Zhang et al. (2020a) who found insomnia in more than one-third of the health care workers working during the COVID-19 pandemic. Burnout can be caused due to insomnia. In fact, Metlaine et al. (2017) stated that job strain represents a burnout risk factor only if associated with insomnia. Banerjee et al. (2020a) in a systematic review of the impact of COVID-19 on psychosocial and mental well-being in the South Asian countries highlighted the increasing stress, anxiety and sleep-related problems in India, especially among the frontliners and health workers. The authors in their advocacy guidance mentioned the need for psychosocial interventions tailored to these needs of the healthcare staff.

Insomnia is a sleep disorder in which one can have trouble falling and/or staying asleep. Good sleep is important for both physical and mental well-being. According to Hess (1965) sleep is “. the expression of a predominance of the trophotropic component of the autonomous nervous system and a preventive measure against exhaustion …” The present-day notion of a circadian rest-activity or sleep-wake rhythm resonates with his concept of alternating trophotropic and ergotropic states. The trophotropic state and the circadian rest state predominantly involve physiological processes that promote energy conservation and restoration as distinguished from the physiological processes and the functional status of the nervous system that help organisms to expend energy (Borbély, 1982; Colten and Altevogt, 2006). During sleep, the arousal systems are shut down allowing the brain to fall asleep. The arousal systems include the thalamus, posterior hypothalamus, neuronal aggregates within the brainstem reticular formation, and basal forebrain. The arousal systems stimulate cortical activation through ascending projections to the cortex and this is characterized by high-frequency gamma and low-frequency rhythmic theta activity. The descending projections to the spinal cord stimulate muscle tonus as well as sensory-motor responsiveness and activity (Jones, 2003). Proper functioning of the arousal systems helps us stay alert and awake. Sleep-wake homeostasis keeps track of the body's requirement of sleep and maintains the sleep-wake cycle.

Stress is a state of disrupted homeostatic balance. It is triggered by intrinsic or extrinsic stressors or situations that are perceived as a threat to one's well-being. The body counteracts by a range of complex physiological and behavioral responses to reestablish eustasis — the optimal body equilibrium (Tsigos et al., 2000). The adaptive stress response involves an intricate network of neuroendocrine, cellular, and molecular infrastructure. Hypothalamic-pituitary-adrenal (HPA) axis and the autonomic nervous system (ANS) work in tandem with other vital centers in the central nervous system (CNS) and tissues/organs in the periphery to yield a successful adaptive stress response. Dysregulation of the stress system can disrupt the body homeostasis leading to a state of cacostasis (adverse effects) or allostasis (achieve stability). Stress and insomnia are not unitary constructs but these two aspects of mental health are intricately intertwined. Sleep and stress response share a common pathway – the hypothalamic-pituitary-adrenal (HPA) axis. Sleep, especially deep sleep, has an inhibitory influence on the HPA axis whereas, activation of the HPA axis can lead to arousal and sleeplessness (Nicolaides et al., 2000). The HPA axis is also responsible for the neuroendocrine adaptation of the stress response (Smith and Vale, 2006). The production of the stress hormone cortisol is triggered by stress-induced activation of the HPA axis. Cortisol is an essential steroid hormone and like many other physiological processes like sleep has a circadian rhythm. In healthy individuals, cortisol levels reach a nadir at midnight and then build up overnight to peak in the morning and then again decline slowly throughout the day. However, when we are under stress the HPA axis gets activated and the adrenal glands release the hormone cortisol into the bloodstream. This prepares the body for the “fight or flight” response which is important for survival. Therefore, on one hand, stress-related activation of the HPA axis might decrease sleep eventually leading to burnout. On the other hand, sleep deprivation can lead to maladaptive changes in the HPA axis and result in neuroendocrine dysregulation. Thus, stress and insomnia might exacerbate each other and create a vicious cycle impacting long term mental health (Basta et al., 2007).

As already discussed, stress and insomnia are common mental health issues among HCWs battling the COVID-19 pandemic in India and the rest of the world. Most studies investigating stress among health care workers have reported global stress scores. Stress, however, is not a unitary construct. It is multifaceted and complex. Various physiological, psychological, social, and emotional factors may contribute to stress. In fact, the items of the PSS-10 were designed to “tap how unpredictable, uncontrollable, and overloaded respondents find their lives” (Cohen et al., 1983). These different aspects of stress might have different predictor variables and might be differently associated with insomnia. Moreover, different components of stress and insomnia might affect different categories of HCWs differently.

In this study, we conducted a factor analysis on the items obtained from the PES-10 and the ISI-7 to investigate the inter-correlation between these measures and extract different factors of these two mental health parameters. We hypothesized that some measures of sleep will significantly relate with stress measures as these two aspects of mental health influence each other. We also hypothesized that different categories of HCWs will score differently on different factors. We expected different socio-demographic and clinical-professional predictors for different factors. Most studies on Indian HCWs have acquired data through online surveys that have inherent limitations such as lack of focus groups and selection bias. To overcome these shortcomings, we conducted a pen and paper survey. Stratified random sampling was attempted to overcome sampling bias.

## Materials and Methods

### Ethics

The study was approved by the institutional ethics committee (DHGMC/2020/349/10). All participants signed an informed consent form approved by the above committee.

### Settings

The study was conducted from 20th April to 20th May at Diamond Harbour Medical College & Hospital (DHGMC), West Bengal, India. During this time COVID-19 was gradually spreading across India thereby mounting pressure on the health care system. DHGMC was converted into a COVID-19 treatment center, well-equipped with an isolation ward, quarantine center, fever clinic, and COVID-19 testing facility.

### Sampling

Approximately, 612 (235 doctors, 259 nurses, 80 ward staff, and 40 non-clinical staff) employees were working at the hospital when this study was carried out. So, the percentages of doctors, nurses, ward staff, and non-clinical staff working during that time were 38.27, 42.18, 13.02, and 6.5%, respectively. We did a stratified random sampling, and the questionnaires were randomly distributed among 308 HCWs (~50% of the total workforce). The 308 HCWs comprised of 118 doctors (38.31%), 130 nurses (42.2%), 40 ward staff (13.0%), and 20 clinical staff (6.5%). Responses were received from only 250 HCWs. Participants having any history of neurological or psychiatric illness were excluded from the study based on self-reports and their scores on the general health questionnaire. After eliminating participants not meeting the inclusion criteria (*n* = 44), incomplete data (*n* = 52), and spurious data (*n* = 14), finally 140 participants were selected for the study. These 140 participants comprised of 56 doctors (40.0%), 46 nurses (32.9%), 20 ward staff (14.3%), and 18 non-clinical staff (12.9%). Thus, the proportion of HCWs included in the final analyses did not match the distribution of HCWs working in the hospital. We, however, did not exclude participants from these final 140 to meet the exact proportion of HCWs working in the hospital as that would have further reduced the sample size. Strict lockdown protocol, social distancing, the growing pressure of COVID-19 patients in the hospital, and the all-pervading fear of death and loss proved to be detrimental for the collection of data, especially through offline forms. HCWs were too preoccupied to focus on research participation. Consequently, we could not follow the stratified random sampling protocol very strictly despite our best efforts.

### Participants

One hundred forty (56 doctors, 46 nurses, 20 ward staff, and 18 non-clinical staff) were selected for the study. Doctors comprised of trained professionals who had at least a bachelor's degree in medicine and surgery (MBBS). Nurses included qualified professionals with at least a diploma in nursing. Ward staff members included trained medical technicians and attendants. Non-clinical staff members included the administrative staff and office workers who were not directly involved in patients' care. All the nurses were females, and all the ward staff members were males (Tables 1, 2).

**Table 1:** Socio-demographic details of the participants.

**Variable name**	**Sample size (*N* = 140)**
Age	37.67 ± 9.847
**Gender**	
Male	61 (43.6%)
Female	79 (56.7%)
**Marital status**	
Married	82 (58.6%)
Unmarried	56 (40.0%)
Separated	2 (1.4%)
**Habitat**	
Urban	84 (60.0%)
Rural	56 (40.0%)
**Education**	
Diploma	2 (1.4%)
Graduate	82 (58.6%)
Postgraduate	56 (40%)
**Family (living with)**	
Children	25 (17.9%)
Parents	63 (45.0%)
Spouse	49 (35.0%)
Single	3 (2.1%)
**Occupation**	
Doctor	56 (40.0%)
Nurses	46 (32.9%)
Ward staff	20 (14.3%)
Non-clinical staff	18(12.9%)
**Media exposure**	
<1 h	14 (10.0%)
<2 h	26 (18.6%)
<3 h	43 (30.7%)
Above 3 h	57 (40.7%)
**Disease**	
None	87 (62.1%)
Diabetes	12 (8.6%)
Hypertension	22 (15.7%)
COPD	11 (7.9%)
Multiple complications	8 (5.7%)

*Key: COPD-Chronic obstructive pulmonary disease*.

**Table 2:** Clinical-professional details of the participants.

**Variable**	**Sample size**	**Stand deviation/**
**name**	**(*N* = 140)**	**percentage**
Duration of Service	10.7	±9.52
**Level of risk of posting**		
Severe risk	35	25.0%
High risk	65	46.4%
Moderate risk	25	17.9%
Low risk	15	10.7%
**Prophylaxis taken**		
Yes	36	25.7%
No	104	74.3%
**Using of mask**		
Always when outdoors	109	77.9%
Even in home	15	10.7%
Only when in workplace	16	11.4%
**Perceived stress severity**		
Low	29	20.7%
Moderate	102	72.9%
High	9	6.4%
**Insomnia severity**		
No (0–7)	73	52.1%
Sub threshold (8–14)	30	21.4%
Moderate (15–21)	24	17.1%
Severe (22–28)	13	9.3%

### Measures

#### Demographic Information

Demographic information was obtained using a customized demographic data sheet. A questionnaire was designed to assess the participant's level of exposure to patients with COVID-19 infection. Based on the information they were categorized into four groups—severe risk (specimen collection unit, and isolation ward), high risk (chest/medicine outdoor, fever clinic, and emergency), moderate risk (specialist outpatient and inpatient department), and low risk (administrative work).

#### The Perceived Stress Scale

The Perceived Stress Scale **(**PSS – 10) (Cohen et al., 1983) has 10 questions/statements and the respondents indicate their levels of agreement (0 = Never; 1 = Almost; 2 = Sometimes; 3 = Fairly Often 4 = Very Often). It includes items measuring reactions to stressful situations as well as measures of stress. The PSS-10 scale has acceptable reliability measures for Indian population (internal consistency-Cronbach's α = 0.731; Spearman-Brown split-half reliability coefficient = 0.71) (Pangtey et al., 2020).

#### Insomnia Severity Index

Insomnia severity index (ISI-7) (Morin et al., 2011) contains seven items that assess the severity of both nighttime and daytime components of insomnia. The first three items assess trouble in initiating, maintaining sleep, and early morning awakening. Other items address dissatisfaction with sleep, daytime functions, recognition of insomnia by others, and finally, distress caused by insomnia. These are scored on a five-point scale ranging from 0 = no problem to 4 = very severe problem. The score of 0–7 depicts the absence of insomnia, 8–14 indicates subthreshold insomnia, 15–21 represents moderate, and 22–28 suggests severe insomnia. ISI has high internal consistency (Cronbach's α = 0.84) test-retest reliability [ICC (2, 1) = 0.84] and validity (correlation with Pittsburgh Sleep Quality Index- *r* = 0.45) for Indian population (Veqar and Hussain, 2020). We have used the original English versions of the above tests as all participants in this study had at least 12 years of formal education.

### Procedure

The participants self-administered the questionnaires at their leisure in their preferred place without the intervention of the researchers. They were requested to return the questionnaires within a week of receiving them. A follow up was initiated if any participant failed to return the questionnaires within the stipulated time. This being a cross-sectional study, the participants responded only once.

### Statistical Analyses

The data were manually entered into Microsoft Excel (Microsoft Corporation, Washington, USA, 2016) after removing all the identifiable information. Statistical analyses were performed using Statistical Package for Social Sciences (SPSS) Statistics for Windows, Version 20.0 (IBM Corp., USA, 2011).

We obtained 17 measures per patient: Insomnia (7 questions) and Perceived Stress (10 questions). A Factor Analysis (FA) using the principal component method with a varimax rotation was conducted on data obtained from 140 patients to reduce the number of variables. It may be noted here that the factor structure of a particular tool may vary due to sampling differences (Gaskin et al., 2017). Existing factor analysis data on PSS-10 are based on samples from different cultures and were collected under different socio-economic and health conditions. So, instead of confirmatory factor analysis based on previous studies, a data-driven approach was taken. “Eigenvalues greater than one” was considered as factor extraction criteria since this is considered to be a reliable technique for factor extraction in exploratory factor analysis (Field, 2009).

Shapiro-Wilk test for normality was done on the total factor scores and it revealed that the data are not normally distributed. After excluding the three outliers (6, 64, and 125) the data conformed to the normality criteria. Hence rest of the analyses were done on these 137 participants.

A mixed-design ANOVA was conducted to test for an interaction between the groups (of HCWs) and the mental health components. This analysis was followed by independent sample *t*-tests to determine how the groups differed across the four mental health components.

Stepwise regression was conducted to test if socio-demographic (age, gender, habitat, marital status, education, family, diseases, and media exposure) and clinical-professional variables (duration of service, quarantine, level of risk, contact with confirmed COVID cases, prophylaxis, and use of mask) could predict the mental health components.

## Results

### Descriptive Analyses

47.9% (67/140) of the HCWs suffered from insomnia. The mean insomnia scores of doctors, nurses, ward staff, and non-clinical staff were 8.7 ± 6.5, 8.1 ± 5.8, 8.9 ± 2.4, and 10.3 ± 5.9, respectively. 79.3% of the HCWs perceived moderate to severe levels of stress. The mean perceived stress scores of doctors, nurses, ward staff, and non-clinical staff were 19.8 ± 4.5, 18.6 ± 4.3, 12.9 ± 3.6, and 16.2 ± 9.5 respectively.

### Group Characteristics

The four groups (Doctors: 34 Male, 22 Female; Nurses: 0 male, 46 Female; ward staff: 20 Male, 0 Female; and Non-clinical staff: 7 Male, 11 Female) were not comparable in age (*p* = 0.006), education (*p* < 001) and gender ratio. Doctors (*M* = 39.23 ± 9.3) and nurses (39.46 ± 11.5) did not have any significant difference in age. The ward staff members had the lowest mean age (31.45 ± 4.8) followed by the non-clinical staff members (35.17 ± 8.4) however, this difference was not statistically significant. The age difference between the nurses and the ward staff members was significant (*p* < 0.05) but, the difference between non-clinical staff members and the nurses was not significant. Doctors were significantly more educated than other health care workers (*p* < 0.05). Nurses, Ward staff, and non-clinical staff did not differ significantly in their levels of education. 64.3% of doctors did not have any comorbidity, 5.4% had diabetes, 23.2% had hypertension, and 7.1% had COPD (chronic obstructive pulmonary disease). Among the nurses, 50% did not have any comorbidity, 15.2% had diabetes, 17.4% had hypertension, and 17.4% had multiple comorbidities. Seventy-five percentage of the ward staff did not have any comorbidity but 25% had COPD. 72.2% of the non-clinical staff did not have any comorbidity, 11.1% had diabetes, 5.6% had hypertension, and 11.1% had COPD. Only 3.6% of the doctors and 5.6% of the non-clinical staff lived alone. The rest of the participants stayed with their families.

Exposure to media was assessed on a scale ranging from 1- (<1 h) to 4 (above 3 h. Mean scores of doctors, nurses, ward staff, and non-clinical staff were 3.3 ± 0.90, 2.5 ± 1.0, 3.3 ± 0.44, 3.2 ± 1.2, respectively. The nurses were significantly less exposed to media compared to other doctors, ward staff, and non-clinical staff. The other three groups did not have any significant differences in media exposure scores. 76.8, 34.7, 50, and 72.2% of the doctors, nurses, ward staff, and non-clinical staff, respectively, were married; 1.7% of doctors and 2.2% of nurses were separated; the rest of the participants were unmarried. 87.5% doctors, 47.8% nurses, 0% ward staff, and 72.2% of the non-clinical staff lived in urban areas. 42.9% doctors, 21.7% of nurses, 0% of ward staff, and 11.1% of non-clinical staff used prophylaxis. All the participants used masks. However, the profuseness of use varied across groups. Seventy-five percentage of doctors, 78.3% nurses, 100% ward staff, and 61.1% of non-clinical staff used masks always when they went out of their home; 21.4% of doctors, 6.5% nurses, and 5.6% of non-clinical staff used masks only while at work; and 3.6% of doctors, 15.2% of nurses, and 33.3% of non-clinical staff used masks even at home. 17.8% of doctors, 6.5%nurses, 100% of ward staff, and 38.9% of non-clinical staff had the habit of smoking. Doctors (12.82 ± 8.4) and nurses (13.1 ± 11.5) did not differ significantly in “duration of service.” The ward staff members (4.5 ± 3.4) and non-clinical staff members (5.6 ± 6.7) did not differ significantly in “duration of service.” However, the doctors and nurses had a greater “duration of service” than the ward staff members and the non-clinical staff members. The level of risk for infection was assessed on a scale ranging from 1– low to 4–Very high. The mean scores of doctors, nurses, ward staff, and non-clinical staff were 3.21 ± 0.76, 2.98 ± 0.72, 2.75 ± 0.44, 1.56 ± 1.1, respectively. There was no significant difference between doctors, nurses, and ward staff in levels of risk for infection, but the non-clinical workers had a significantly lower risk for infection compared to the other three groups (*p* < 0.05). Among the participants, 17.9% of doctors, 15.2% of nurses and 5.6% of non-clinical staff members were quarantined. None of the ward staff was quarantined.

### Factor Analysis

#### The Initial Factor Analysis

A factor analysis with the principal component method was conducted on the 17 measures that were obtained from the ISI-7 and PSS-10. KMO value indicated that the sample was factorable (KMO = 0.768). Homogeneity of variance was confirmed by Bartlett's test [*x*^2^ (136) = 926.7, *p* < 0.001]. The diagonals of the anti-image correlation matrix were over 0.5 for all items except the PSS (Q4). This item was dropped from the final analysis.

#### The Final Factor Analysis

The final factor analysis was done on 16 items. KMO of the final model was 0.786 and Bartlett's test was significant [*x*^2^ (120) = 877.4, *p* < 0.001] confirming that the data were factorable (Field, 2009). The diagonals of the anti-image correlation matrix were above 0.5 for all items. Communalities were above 0.5 for all items in the final analysis except P-6 (0.42). We extracted four factors with eigenvalues above 1. The four components explained 29.6, 16.0, 10.0, and 6.6% of the variance, respectively. The cumulative percentage of variance explained by the five components was 62.2%. The rotated component matrix with the communalities of the items is given in Table 3. After scrutinizing the individual items of these four factors, we named them: (1) Insomnia (2) Stress-related Anxiety (3) Stress-related Irritability, and (4) Stress-related Hopelessness. Hereafter these four factors will be referred to as Insomnia, Anxiety, Irritability, and Hopelessness, respectively. Factor *hopelessness* had less than three-item loadings, but we retained it as a separate factor because irritability and hopelessness are different aspects of stress. Further analyses were done on these four factor-scores.

**Table 3:** Rotated component matrix.

**Items**	**Sleeplessness**	**Anxiety**	**Irritability**	**Hopelessness**	**Communalities**
Insomnia_6	0.872				0.778
Insomnia_7	0.85				0.735
Insomnia_5	0.792				0.697
Insomnia_2	0.768				0.651
Insomnia_4	0.651				0.563
Insomnia__1	0.618				0.522
Insomnia_3	0.602				0.502
PS_1		0.835			0.711
PS_3		0.792			0.654
PS_2		0.737			0.589
PS_9		0.721			0.655
PS_5			0.84		0.697
PS_7			0.74		0.735
PS_8			0.705		0.544
PS_10				0.725	0.59
PS_6				0.517	0.423

*PS, Perceived stress; INS, Insomnia; SUP, Stress-due-to-unpredictability; SOL, Stress-due-to-overload; SUC, Stress-due-to-uncontrollability*.

### Hypothesis Testing

After factor analysis, factor scores were scanned for outliers. Shapiro-Wilk test for normality was done on the total factor scores and it revealed that the data are not normally distributed. After excluding the three outliers (6, 64, and 125) the data conformed to the normality criteria. Hence rest of the analyses were done on these 137 participants.

These 137 participants were divided into four groups based on their profession. There were 55 doctors (Age: *M* = 39.22 ± 9.3, 33 Male and 22 Female), 45 nurses (Age: *M* = 39.60 ± 11.6; 0 Male and 45 Female), 20 ward staff (Age: *M* = 31.45 ± 4.8; 20 Male and 0 Female), and 17 non-clinical staff (Age: *M* = 34.06 ± 7.2; 6 Male and 11 Female).

**Figure 1:**
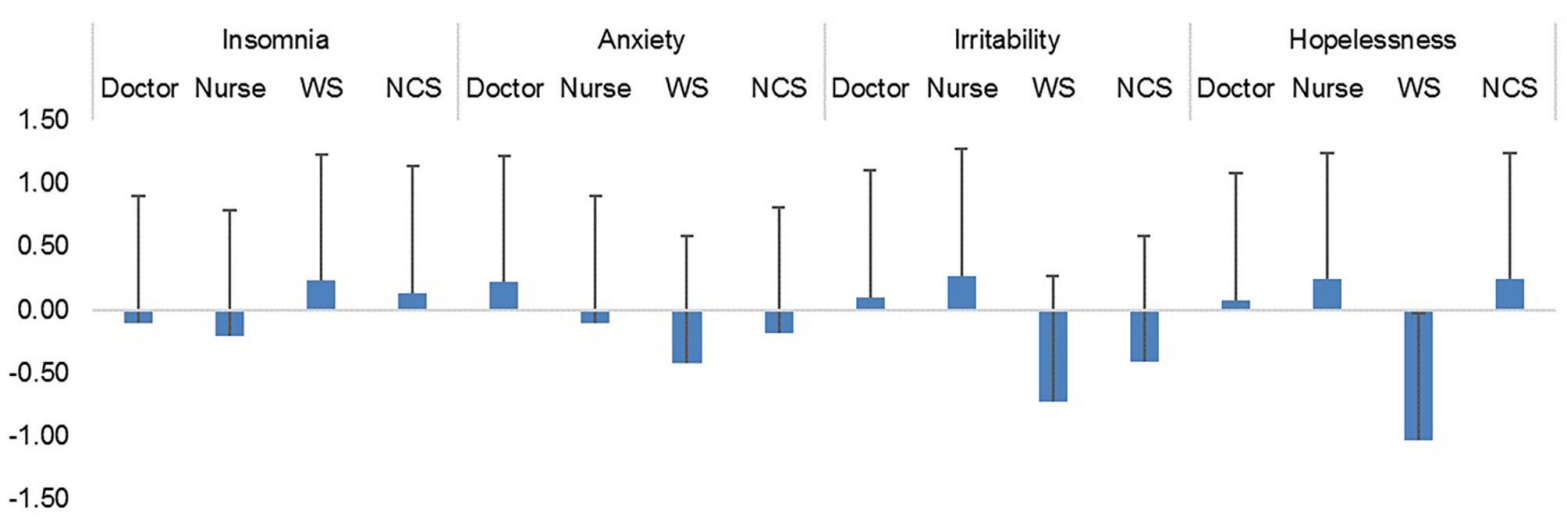
Mean scores of different categories of health care workers.

The mixed design ANOVA was carried out with groups of HCWs (Doctor, *N* = 55; Nurse, *N* = 45; Ward staff (WS), *N* = 20; and Non-Clinical staff (NCS), *N* = 17) as a between-subject variable and the four mental health components obtained from the factor analysis (Insomnia, Anxiety, Irritability, and Hopelessness) as a within-subject variable. The test did not yield any significant main effect of mental health factors [*F*_(3, 399)_ = 0.84, *p* = 0.47, observed power = 0.24]. However, there was a significant main effect of group [*F*_(3, 133)_ = 9.7, *p* < 0.001; observed power = 0.99] and significant Factor scores x Group interaction [*F*_(9, 399)_ =3.63, *p* < 0.001; observed power = 0.99]. Thus, different categories of HCWs responded differently to the different mental health factors (Figure 1).

### Mean Scores of Healthcare Workers in Four Components of Mental Health

Independent sample *t*-tests revealed that compared to the ward staff, doctors were significantly more anxious (*p* = 0.005), irritable (*p* < 0.001), and hopeless (*p* = 0.001). Nurses were more irritable (*p* < 0.001), and hopeless (*p* < 0.001) than the ward staff. Doctors were more irritable than the non-clinical staff (*p* = 0.027). Nurses were also more irritable than the non-clinical staff (*p* = 0.010).

Non-clinical staff members were more hopeless than the ward staff (*p* = 0.008). Ward staff members experienced more insomnia than the nurses (*p* = 0.01). There were no significant differences between the doctors and the nurses (Table 4).

**Table 4:** Result of independent sample *t*-tests.

**Factor**	**Group**	**Mean**	**(SD)**	**Pairs compared**	***T*-value**	**df**	***P*-value**	**Cohen's *d***
Insomnia	Doctor	−0.10	(1.10)	Doctor - Nurse	0.528	98	0.599	0.107
	Nurse	−0.21	(0.94)	Doctor - WS	−1.923	72.989	0.058	0.399
	WS	0.23	(0.39)	Doctor - NCS	−1.179	53.143	0.244	0.273
	NCS	0.14	(0.57)	Nurse - WS	−2.66	62.785	0.01^*^	0.611
				Nurse - NCS	−1.422	60	0.16	0.450
				WS - NCS	0.578	35	0.567	0.184
Stress-related	Doctor	0.22	(0.83)	Doctor - Nurse	1.715	85.199	0.09	0.346
anxiety	Nurse	−0.10	(1.01)	Doctor - WS	2.917	73	0.005^*^	0.752
	WS	−0.42	(0.87)	Doctor - NCS	1.184	19.992	0.25	0.358
	NCS	−0.18	(1.34)	Nurse - WS	1.23	63	0.223	0.339
				Nurse - NCS	0.267	60	0.79	0.067
				WS - NCS	−0.625	26.696	0.537	0.531
Stress-related	Doctor	0.10	(1.05)	Doctor - Nurse	−0.833	98	0.407	0.168
irritability	Nurse	0.27	(0.96)	Doctor - WS	4.131	55.736	0.000^*^	0.724
	WS	−0.73	(0.64)	Doctor - NCS	2.299	39.219	^*^^1^0.027^*^^1^	0.566
	NCS	−0.41	(0.72)	Nurse - WS	4.939	53.303	<0.001^*^	1.22
				Nurse - NCS	2.66	60	0.01^*^	0.801
				WS - NCS	−1.418	35	0.165	0.469
Stress-related	Doctor	0.08	(0.79)	Doctor - Nurse	−1.224	96.425	0.224	0.232
hopelessness	Nurse	0.24	(0.57)	Doctor - WS	3.914	25.556	0.001^*^	1.111
	WS	−1.03	(1.17)	Doctor - NCS	−0.418	18.623	0.681	0.129
	NCS	0.24	(1.56)	Nurse - WS	4.626	23.037	<0.001^*^	1.380
				Nurse - NCS	0.007	17.627	0.994	0.000
				WS - NCS	−2.832	35	0.008^*^	0.921

WS, Ward staff; NCS, Non-clinical staff; SD, Standard deviation; df, Degree of freedom;

* Statistically significant after FDR correction;

*1 *Statistically not significant after FDR correction*.

### Exploratory Analyses

Stepwise linear regression with the socio-demographic variables (age, gender, habitat, marital status, education, family, diseases, and media exposure) as predictors were conducted for all the four factors (Insomnia, anxiety, irritability, and hopelessness). Age (β = −0.431, *t* = −6.1, *p* < 0.001), education (β = 0.358, *t* = 4.4, *p* < 0.001), gender (β = 0.202, *t* = 2.7, *p* = 0.008), and habitat (β = −0.201, *t* = −2.6, *p* = 0.011) predicted anxiety [*F*_(4, 132)_ = 18.27, *p* < 0.001, *R*^2^ = 0.356, Cohen's *f*^2^= 0.552] indicating lower age, higher education, female gender and urban habitat were associated with higher anxiety. Age (β = 0.480, *t* = 6.3, *p* < 0.001) and marital status (β = 0.247, *t* = 3.2, *p* = 0.002) predicted irritability [*F*_(2, 134)_ =22.3, *p* < 0.001, *R*^2^ = 0.249, Cohen's *f*^2^ = 0.331]. Older age and single marital status predicted irritability. Gender (β = 0.412, *t* = 5.2, *p* < 0.001), marital status (β = −0.203, *t* = −2.5, *p* = 0.012) and disease (β = 0.175, *t* = 2.3, *p* = 0.025) predicted hopelessness [*F*_(3, 133)_ = 11.4, *p* < 0.001, *R*^2^ = 0.205, Cohen's *f*^2^ = 0.257]. Female gender, married status, and higher number of ailments contributed to perceived hopelessness. None of these variables predicted insomnia (Table 5).

**Table 5:** Results of stepwise linear regressions with the demographic variables.

**Dependent variable**	**Predictors**	**β**	***t*-value**	***p*-value**	***F*-value**	**df**	***p*-value**	***R*-square**	**Cohen's(f2)**
Insomnia	None	–	–	–	–	–	–	–	–
Stress-related	Age,	−0.431	−6.084	0.000	18.268	4,132	0.000	0.356	0.552
anxiety	Education	0.358	4.384	0.000					
	Gender	0.202	2.7	0.008					
	Habitat	−0.201	−2.574	0.011					
Stress-related	Age	0.48	6.316	0.000	22.257	2,134	0.000	0.249	0.331
irritability	Marital-status	0.247	3.241	0.002					
Stress-related	Gender	0.412	5.177	0.000	11.428	3,133	0.000	0.205	0.257
hopelessness	Marital-status	−0.203	−2.55	0.012					
	Disease	0.175	2.262	0.025					

Stepwise linear regression with the clinical-professional variables (duration of service, quarantine, level of risk, contact with confirmed COVID cases, prophylaxis, and use of mask) as predictors were conducted for all the four factors (insomnia, anxiety, irritability, and hopelessness). Quarantine (β = −0.206, *t* = −2.4, *p* = 0.016) significantly predicted insomnia [*F*_(1, 135)_ = 5.95, *p* = 0.016, *R*^2^ = 0.042, Cohen's *f*^2^ = 0.043]. People who were quarantined were more prone to suffer from insomnia. Duration of service (β = −0.467, *t* = −5.88, *p* < 0.001) and use of prophylaxis (β = −0.197, *t* = −2.5, *p* = 0.015] predicted anxiety [*F*_(2, 134)_ = 17.78, *p* < 0.001, *R*^2^ = 0.210 Cohen's *f*^2^ = 0.265]. Fewer years in service and use of prophylaxis was associated with anxiety. Duration of service (β = 0.462, *t* = 6.45, *p* < 0.001), quarantine (β = −217, *t* = −2.98, *p* = 0.003] and level of risk (β = −0.165, *t* = −2.3, *p* = 0.024) predicted irritability [*F*_(3, 133)_ = 21.58, *p* < 0.001, *R*^2^ = 0.327, Cohen's *f*^2^ = 0.485]. Greater duration of service, quarantine, and a greater level of risk contributed to irritability. None of these variables predicted hopelessness (Table 6).

**Table 6:** Results of stepwise linear regressions with clinical-professional variables.

**Dependent variable**	**Predictors**	**β**	***t*-value**	***p*-value**	***F*-value**	**df**	***p*-value**	***R*-square**	**Cohen's(f2)**
Insomnia	Quarantine	−0.206	−2.44	0.016	5.956	1,135	0.016	0.042	0.043
Stress-related	Duration of service	−0.467	−5.878	0.000	17.777	2,134	0.000	0.21	0.265
anxiety	Prophylaxis	−0.197	−2.474	0.015					
Stress-related	Duration of service	0.462	6.453	0.000	21.581	3,133	0.000	0.327	
irritability	Quarantine	−0.217	−2.983	0.003					0.485
	Level of risk	−0.165	−2.277	0.024					
Stress-related	None	–	–	–	–	–	–	–	–
hopelessness									

## Discussion

Our study aimed to investigate the different components of perceived stress and insomnia experienced by the HCWs and how different socio-demographic and clinical-professional factors influenced these components. The factor analysis of insomnia and stress scales yielded four factors which were identified as – (1) Insomnia, (2) Stress-related Anxiety, (3) Stress-related Irritability and (4) Stress-related Hopelessness. The four factors explained 62.2% of the variance. Perceived stress yielded three factors and this is consistent with Pangtey et al. (2020) who validated the Hindi version of PSS-10 in the adult urban population of Delhi.

All the 7 questions of the insomnia scale loaded on the first factor. Insomnia was found to be the most important factor and it explained 29.6% of the variance. There was no significant correlation between the insomnia factor and the other three factors of perceived stress. This is consistent with Gupta et al. (2020) who found no significant differences in perceived stress among three different groups with varying levels of nighttime sleep duration after lockdown due to COVID-19. It may be noted that insomnia can be caused by several other factors apart from stress. In this study, quarantine significantly predicted insomnia. More screen time, reduced physical activity, change in daily routine, and staying away from home in a quarantine center could contribute to insomnia. Concern for one's own health, apprehensions for their loved ones, financial worries, etc. could exacerbate anxiety and stress during the quarantine. In response to the stress the cortisol level may shoot up and disrupt the sleep-wake cycle increasing sleep fragmentation, dreaming and insomnia (Basta et al., 2007). Similarly, the blue-wavelength light from the electronic screen may force the brain into confusing between day-night cycle and suppress the production of the sleep hormone melatonin (Tähkämö et al., 2019). Reduced physical activity (PA) may decrease total energy expenditure and affect sleep quality. Exercise is reported to significantly decrease REM sleep (Wang and Boros, 2019) thereby expounding the mechanism of PA effect on sleep. Prevalence of Insomnia was quite high (49.7%) among the HCWs who participated in this study. This percentage is slightly higher than that reported by Lai et al. (2020) and Bhargava et al. (2020). Ward staff members were most likely to experience insomnia. Compared to doctors and nurses, other HCWs were more prone to suffer from insomnia. Smoking could be the possible reason for the elevated insomnia scores in these groups. One-hundred percentage of the ward staff members and 38.9% of the non-clinical staff members had the habit of smoking. The percentages of smokers among the doctors and nurses were much lower. The stimulating effect of nicotine may prevent smokers from falling asleep and later on as night evolves they may have sleep disturbance due to withdrawal from nicotine (Zhang et al., 2008).

Stress due to unpredictability has been referred to as “*anxiety”* in this study. HCWs with lower age, higher education, female gender, and urban habitat experienced higher levels of anxiety. In fact, doctors who formed the most educated group among the HCWs were the most anxious of all. As we have seen in several patients, better knowledge and understanding of the disease can engender stress and anxiety (Selinger et al., 2013; Zhang et al., 2014). Doctors are not an exception to this rule. Female HCWs and HCWs with lower age experienced greater anxiety. This is in line with Matud (2004) who reported significantly more stress in women even after adjusting for sociodemographic variables. In fact, our result is consistent with studies that report sexual dimorphism in stress reactivity and increased female vulnerability to stress-related disorders (Bangasser and Wicks, 2017; Novais et al., 2017). For example, research reports that female sex hormones attenuate the sympathoadrenal and HPA responsiveness leading to sluggish cortisol feedback on the brain and less or delayed containment of the stress response (Verma et al., 2011). Moreover, human female hypothalami have increased corticotropin-releasing hormone (CRH) content relative to male hypothalami and plasma adrenocorticotropin hormone responses to the ovine CRH are found to be significantly greater among women as compared to men (Gallucci et al., 1993). Consequently, women have greater sensitivity and lower tolerance to negative emotions and are reported to have two to three times higher risk of developing post-traumatic stress symptoms than men (Kessler et al., 2005; Tolin and Foa, 2006). Our results are also in line with the American Psychological Association (APA)'s report of 2019 ( Stress in America 2013, Are Teens Adopting Adults' Stress Habits? 2013), which states that younger adults and women are more stressed out. This is partly consistent with Remes et al. (2016) who stated that the prevalence of anxiety disorder is higher in women and young adults. However, it may be noted that anxiety referred to here is an aspect of stress and we have not used any tool to measure anxiety *per se*. Nonetheless, these two psychobiological states are reported to have neural as well as behavioral overlaps (Daviu et al., 2019). Our result is consistent with several other studies that report higher levels of stress in people living in cities compared to rural areas (Srivastava, 2009; Gruebner et al., 2017). Fewer years in service and use of prophylaxis was associated with anxiety. HCWs with junior titles were probably less adapted to handle such crises and consequently had higher levels of stress. Higher stress levels could result from the use of prophylaxis (Juurlink, 2020). Additionally, people who are more stressed could be more inclined to use prophylaxis.

Stress due to overload has been referred to as “*irritability*”. Doctors and nurses scored high on this factor compared to other HCWs. This is consistent with recent studies examining the mental health status of HCWs during COVID-19 (Lai et al., 2020). Older and single HCWs were more irritable. This result is quite intuitive. Older people are more likely to succumb to tiredness due to overwork and single HCWs were probably more stressed because they were handling their emotional and physical burden single-handedly. The result is consistent with a recent study that found lower levels of stress hormones in healthy married adults (Chin et al., 2017). Greater duration of service, quarantine, and a greater level of risk contributed to irritability. This result again is quite expected. Greater duration of service indicates higher age and as already explained older people might capitulate to fatigue and exhaustion more easily than younger people. Moreover, apart from emotional turmoil, quarantine might impose a physical burden as well. Middle-class salaried Indians usually have the privilege of domestic help to take care of household chores. Quarantine could inadvertently repeal this privilege thereby escalating unwonted physical burden and hence stress. This is partly consistent with a study in the general population (Stress, Stigma and Sleep loss: COVID-19 Takes a Heavy Toll on mental Health- The New Indian Express, 2020) that was covered by the *New Indian Express*. HCWs posted in specialties such as a respiratory ward, infectious-diseases ward, or critical-care ward, where there is a high risk, are plausibly sharing the greatest workload during this pandemic. Consequently, they are probably under greater stress than other HCWs. Wearing the heavy PPE in this hot and humid climate might add to their distress which has been highlighted among the physicians in India repeatedly during the pandemic (Banerjee et al., 2020b).

Stress due to uncontrollability has been denoted as “*hopelessness”* in this study. Female gender, single marital-status, and greater ailments contributed to perceived hopelessness. Ward staff members were found to be the most hopeful among the HCWs. Incidentally, all the ward staff members were males. This is in line with the linear regression result that indicated gender as the most important predictor of perceived hopelessness. Female HCWs were more likely to be perturbed with the feeling of hopelessness. Our result is consistent with studies that report a feeling of powerlessness among HCWs. Females, being more empathetic, are perhaps more likely to feel hopeless when they witness people suffering and dying. Our findings are also in line with Podder et al. (2020), who reported higher levels of perceived stress in female physicians. In contrast to irritability, married HCWs were found to be more hopeless. Concern for family members and their well-being could contribute to their feeling of hopelessness. The result is somewhat similar to Hacimusalar et al. (2020), who found that the proportion of people who reported increased anxiety was significantly higher in married people compared to single ones. The authors also reported that increase in anxiety levels explained 28.9% of the increase in hopelessness levels. HCWs with a greater number of ailments had greater perceived hopelessness. Numerous scientific journals and social media platforms are continuously reporting that patients with lung diseases, diabetes, and heart diseases are at increased risk for severe complications from COVID-19 (Guan et al., 2020a,b; Sanyaolu et al., 2020). This awareness and a focus on the uncontrollable could worsen the feeling of hopelessness in HCWs with these ailments (Lai et al., 2020).

In sum, this study revealed that the HCWs working in India during the first phase of the pandemic experienced significant mental health symptoms. Several factors contributed to their psychological distress. Most of these factors such as higher age, female gender, higher education, urban habitat, single status, having comorbidities, longer duration of service, a greater level of risk, and quarantine were found to affect the mental health status of HCWs from other countries as well (Vindegaard and Benros, 2020). Quarantine emerged as the predictor of insomnia and this is consistent with several other studies that reported “sense of isolation” as a relevant stressor in quarantined HCWs (Carmassi et al., 2020). However, in this study perceived Stress was considered as a multidimensional construct and the three different components of perceived stress were found to have different predictive factors. In some cases, the factors were differently correlated with different components of perceived stress. For example, age and duration of service were negatively correlated with stress-related anxiety but positively correlated with stress-related irritability. Similarly, while single status predicted irritability, married status predicted hopelessness. The result emphasizes the pressing need to look beyond the global (perceived stress) scores. As in several other studies (Buselli et al., 2020), female HCWs were found to have higher stress-related anxiety and hopelessness. Doctors and nurses had higher levels of stress-related anxiety and irritability. The results of this study make a case for personalized mental health care for HCWs working in different capacities and under different circumstances.

## Limitations and Future Directions

Small sample size, sampling from a particular region of India, cross-sectional design, and unequal and disproportional groups limit the scope of generalizability of the findings of this study. Albeit we have applied FDR (false discovery rate) correction for the *t*-tests and reported effect sizes to reveal the strength of the statistical results, multiplicity of testing is another factor that might affect the statistical power of the tests conducted. Moreover, this study might not represent the mental health issues of HCWs working across India or throughout the world. Culturally diverse populations having different psychological make-ups may respond differently in similar situations. For example, while the study from Kashmir (Khanam et al., 2020) reported higher levels of stress among male HCWs, we found the female HCWs more stressed. Different socio-political situations in these two states of India could be responsible for these contrasting results. The female employment rate in Jammu and Kashmir is abysmally low (7.9%) compared to that of West Bengal (20.5%) (Agarwal, 2018) from where the data was collected for the present study. Kashmiri women who finally get to join the workforce after braving the adverse socio-political situation are perhaps psychologically stronger and more resilient than Bengali women who enjoy a relatively safe and liberal environment. Socio-cultural differences therefore might influence the intensity and modulate the predictive factors of mental health components. So, in order to strategically target therapeutic interventions and to establish the possible impact of the pandemic on the mental health of HCWs, confirmation with a larger sample size covering diverse populations will be an important next step. Since this study is cross-sectional it has predictive limitations as exposure and outcome have been assessed simultaneously. Well-designed longitudinal studies in the future might help track the long-term effects of the pandemic on the mental health of HCWs. Further, qualitative studies grounded in the perspectives of healthcare workers and their perceived challenges during COVID-19 will have important implications for policy changes related to their welfare and safety. However, despite these limitations, the results of this work appear to be substantially in line with previous studies investigating the impact of Covid-19 on the mental health of HCWs. For example, gender differences in the prevalence of stress-related symptoms and quarantine as a predictor of higher stress levels in HCWs have been reported in previous studies (Buselli et al., 2020; Carmassi et al., 2020) that investigated HCWs from other parts of the world. Considering the paucity of research on mental health issues of HCWs fighting COVID 19 in India, this study investigates important and interesting data which will help lend deeper insight into the problems of the HCWs working in different socio-cultural environments.

## Conclusion

The study revealed that the HCWs were working with enormous stress and sleep difficulty during the early phase of the pandemic. Different categories of HCWs were affected differently on different factors of perceived stress. While doctors scored higher on stress-related anxiety, nurses scored higher on stress-related irritability, and both nurses and non-clinical staff members scored high on stress-related hopelessness. Different factors modulated insomnia, stress-related anxiety, stress-related irritability, and stress-related hopelessness. For example, duration of service, and use of prophylaxis predicted stress-related anxiety, while the duration of service, quarantine, and level of risk predicted stress-related irritability. More importantly, the duration of service was negatively correlated with stress-related anxiety but positively correlated with stress-related irritability. Thus, this study emphasizes the fact that perceived stress is a multifactorial construct, and reporting global perceived stress scores might result in an oversimplification of the complex and intricate psychological disorder. Impoverished assessment may subsequently lead to inadequate and inappropriate treatment plans. Personalized treatment for different categories of HCWs should be maneuvered appropriately to grapple with the mental health issues of the HCWs in this difficult time. Advanced healthcare work-place strategies and tailored policies will help fight the stress and preserve this “frontline workforce” during the COVID-19 and post-pandemic aftermath.

## Data Availability Statement

The raw data supporting the conclusions of this article will be made available by the authors, without undue reservation.

## Ethics Statement

The studies involving human participants were reviewed and approved by DGHMC, West Bengal University of Health Sciences. The patients/participants provided their written informed consent to participate in this study.

## Author Contributions

SeC: concept, design, data collection, data curation, data interpretation, drafting the manuscript, and reviewing and editing. MC: statistical analyses, data interpretation, data visualization, drafting the original manuscript, and editing and revising it critically for important intellectual content. DB: concept, literature review, data curation, drafting the manuscript, organization, reviewing and editing, and revising. SG: design, supervision, editing, and reviewing. ShC: concept, design, data collection, and data preprocessing. UD: design, supervision, editing, and reviewing. All authors have read and approved the final version of the manuscript.

## Conflict of Interest

The authors declare that the research was conducted in the absence of any commercial or financial relationships that could be construed as a potential conflict of interest.
